# Examining frailty phenotypes of community-dwelling older adults in Taiwan using the falls risk for older people in the community – Taiwan version (Tw-FROP-Com)

**DOI:** 10.18632/aging.206231

**Published:** 2025-04-01

**Authors:** Ya-Mei Tzeng, Senyeong Kao, Wun-Sin Chen, Shueh-Fen Chen, Shan-Ru Li, Yu-Lung Chiu, Yu-Tien Chang, Yaw-Wen Chang

**Affiliations:** 1 School of Public Health, National Defense Medical Center, Taipei City, Taiwan; 2 Institute and Department of Physiology and Biophysics, National Defense Medical Center, Taipei City, Taiwan; 3 Department of Senior Citizen Service Business, St. John’s University Taipei Campus, New Taipei City, Taiwan; 4 Department of Family and Community Medicine, Tri-Service General Hospital, National Defense Medical Center, Taipei City, Taiwan; 5 School of Medicine, National Defense Medical Center, Taipei City, Taiwan

**Keywords:** frailty, fall risk, fried frailty criteria, older adults, Tw-FROP-Com

## Abstract

Background: Falls are the second leading cause of accidental injury-related deaths among Taiwanese adults aged 65 and older. This study examined the association between Fried frailty phenotypes and fall risk in this population.

Materials and Methods: A cross-sectional study was conducted in Keelung City with 375 participants from an Elderly Fall Prevention Program. Frailty was assessed using the modified Fried criteria: weakness, slowness, exhaustion, low physical activity, and unintentional weight loss. Participants with 0–2 criteria were classified as non-frail, and those with 3 or more as frail. Fall risk was evaluated using the Taiwan version of the Falls Risk for Older People in the Community (Tw-FROP-Com), a 28-item tool scoring 0–60 across 13 risk factors.

Results: Participants had a mean age of 75.4 ± 6.8 years; 76.0% were female, 18.7% were frail, and 32.7% had fallen in the past year. Those with a fall history had higher rates of weakness (56.7%), slowness (49.6%), and frailty (26.1%). Regression analysis showed that weakness (β = 0.64), slowness (β = 0.21), exhaustion (β = 1.28), unintentional weight loss (β = 3.99), and low physical activity (β = 0.88) were significantly associated with increased fall risk. Frailty explained over 50% of fall risk variance, with unintentional weight loss as the strongest predictor.

Conclusion: Unintentional weight loss is the most significant predictor of fall risk among frailty traits. Individual frailty components better predict fall risk than composite frailty measures.

## INTRODUCTION

Falls are the second leading cause of death from unintentional injuries worldwide [1] and among adults aged 65 and older in Taiwan. Globally, over 80% of the 684,000 annual fall-related deaths occur in low- and middle-income countries. Adults aged 60 and older have the highest rates of fatal falls, with approximately 37.3 million falls each year severe enough to require medical attention. In Taiwan, one in six adults aged 65 and older has experienced a fall, and one in twelve has sought medical care due to fall-related injuries [2].

Falls among older adults often lead to functional decline due to disability, increasing their dependence on others and raising the risk of becoming a burden on caregivers or facing premature admission to nursing homes [3]. Additionally, falls can significantly impact physical and mental health, social functioning, and overall quality of life [4]. In response to these challenges, the World Health Organization (WHO) emphasizes the need for comprehensive fall prevention strategies, including research, education, training, safer environments, and policy development aimed at reducing fall risk [5].

Many factors contribute to falls suffered by older people, such as frailty [6], balance dysfunction, mobility problems, impaired vision, lack of vitamin D, fear of falling, depression, side effects of specific medications, and the presence of home hazards [7]. Frailty is a clinical condition resulting from age-related declines in physiological reserves. Consequently, frail older adults, including persons who have entered the prefrail stage, are likely to experience recurrent falls [7, 8]. Recently, frailty in the older population has attracted much attention because of studies showing its link to negative outcomes such as falls, institutionalization, hospitalization, incident disability, and death [9, 10]. Notably, the frailty phenotype has been associated with a greater risk of falls in women aged 55 and older [11]. A prospective cohort study of older men indicated that a simple frailty index—comprising weight loss, the inability to rise from a chair, and poor energy (Study of Osteoporotic Fractures index)—predicted the risk of falls, disability, fractures, and mortality as effectively as the more complex Cardiovascular Health Study (CHS) index. Components of the CHS include unintentional weight loss, poor grip strength, poor energy, slowness, and a low level of physical activity [12]. Another longitudinal cohort study used the CHS index and the Women’s Health Initiative (WHI) phenotypes to predict fall risk in older women. Higher fall rates (Hazard Ratio (HR) = 1.48, *p* = 0.003) were observed in the WHI frailty phenotype group compared with the CHS group [13]. The WHI phenotype, which does not require direct physical performance measurements, may be useful for the general older population [13]. In Japan, a cross-sectional study examined the prevalence and associated factors of cognitive frailty and cognitive frailty-related falls in community-dwelling older adults. Age, chronic disease, the Timed Up and Go Test, and the Council on Nutrition Appetite Questionnaire were significantly associated with cognitive frailty and cognitive frailty-related falls [14].

Numeric frailty assessment tools have been developed and have demonstrated their ability to predict the risk of falling. However, there is no gold standard for assessing frailty [15]. Currently, a frailty index that does not require a physical examination is needed to predict fall risk and guide preventive interventions for the broader older population. Simple measurement tools can assess frailty as accurately as complex ones. Fried’s frailty phenotypes meet these criteria and have been widely adopted in several studies [16–18].

According to Fried, frailty comprises five elements: weakness, slowness, exhaustion, low physical activity, and unintentional weight loss [19]. In most studies on frailty and fall risk, frailty has been treated as a categorical scale, such as non-frail/frail or non-frail/pre-frail/frail [20–23]. However, few studies have examined the correlation between individual frailty phenotypes and fall risk in Taiwan. Moreover, it remains unclear which frailty phenotype contributes most to the fall risk among community-dwelling older adults in Taiwan.

For fall risk assessment, we used the Falls Risk for Older People in the Community (FROP-Com), which has been widely applied in various older populations worldwide [24–27]. FROP-Com is simple to use and does not require physical measurements. As the first research team to translate and validate the FROP-Com into the Taiwan version (Tw-FROP-Com), our study effectively evaluated fall risk among Taiwanese older adults [28]. Therefore, this study aimed to explore the relationship between Fried’s frailty phenotype and fall risk among community-dwelling older adults in Taiwan using the Tw-FROP-Com.

## RESULTS

Of the 425 subjects screened for a high risk of falling, only 375 participants who completed the frailty and fall risk assessments were included in the analysis. The sociodemographic characteristics of the participants and their fall risk are presented in Table 1. Participants had a mean age of 75.4 ± 6.8 years, were predominantly women (76.0%), and the majority had a low level of education (23.7% were illiterate with no formal education, and 46.5% had only an elementary school education, totaling 70.2%). Additionally, most participants were from the Hokkien ethnic group (85.1%), lived with a spouse (55.7%), lived with others (83.9%), were cared for by others (59.6%), and had no history of falls (67.3%). Regarding frailty components, most participants did not exhibit weakness (52.4%), slowness (61.1%), exhaustion (74.3%), or low physical activity (20.0%).

**Table 1:** Univariate analysis of basic demographic characteristics and fall risk among participants (*n* = 425).

**Variables**	***N* (%)**	**Tw-FROP-Com, The risk of falls, (Mean ± SD)**	***p*-value**
**Gender**			
Male	102 (24.0)	5.24 ± 4.07	0.388
Female	323 (76.0)	4.86 ± 3.74	
**Age**			
65–74 years old	211 (49.6)	4.32 ± 3.58	0.001^**^
≥75 years old	214 (50.4)	5.57 ± 3.95	
**Educational level**			
Illiterate	97 (23.7)	5.89 ± 4.27	0.022^*^
Elementary school	190 (46.5)	4.61 ± 3.55	
Junior high school and higher	122 (29.8)	4.76 ± 3.79	
**Race**			
Hokkien	354 (85.1)	4.98 ± 3.85	0.898
Non-Hokkien	62 (14.9)	5.05 ± 3.85	
**Marital status^a^**			
With a spouse	233 (55.7)	4.48 ± 3.53	0.007^**^
Without a spouse	185 (44.3)	5.49 ± 4.05	
**Live with others or not**			
Live with others	355 (83.9)	4.86 ± 3.87	0.277
Live alone	68 (16.1)	5.41 ± 3.51	
**Caregivers**			
Cared by others	251 (59.6)	4.96 ± 3.83	0.915
Cared by themselves	170 (40.4)	4.92 ± 3.83	
**History of fall**			
Falling in the past year	139 (32.7)	7.85 ± 3.68	<0.001^**^
No	286 (67.3)	3.54 ± 3.01	
**Frailty component**			
** *Weakness n* = (*411*)**			
Yes	197 (47.6)	6.08 ± 4.21	<0.001^***^
No	217 (52.4)	3.83 ± 3.02	
** *Slowness* (*n = 409*)**			
Yes	159 (38.9)	6.92 ± 4.10	<0.001^***^
No	250 (61.1)	3.50 ± 2.62	
** *Exhaustion* (*n = 408*)**			
Yes	105 (25.7)	6.50 ± 4.07	<0.001^***^
No	303 (74.3)	4.40 ± 3.54	
** *Weight loss* (*n = 395*)**			
Yes	18 (4.6)	10.28 ± 4.51	<0.001^***^
No	377 (95.4)	4.77 ± 3.64	
** *Low activity* (*n = 420*)**			
Yes	84 (20.0)	6.40 ± 4.62	0.001^**^
No	336 (80.0)	4.57 ± 3.49	
**Frailty status^b^ (*n* = 375)**			
Yes	70 (18.7)	8.70 ± 4.13	<0.001^***^
No	305 (81.3)	4.07 ± 3.00	

^a^“With a spouse” includes married status; “Without a spouse” includes single, divorced, and widowed. ^b^Frailty status was assessed based on Fried’s phenotype criteria. Participants meeting ≥3 of 5 criteria were classified as frail; those with 0–2 were non-frail. One-way ANOVA was used for comparisons involving more than two groups; independent sample t-tests were used for comparisons between two groups. ^*^p < 0.05, ^**^p < 0.01, ^***^p < 0.001.

We used the Tw-FROP-Com to evaluate fall risk. There was no significant difference in Tw-FROP-Com scores between males and females (*p* = 0.388). Participants over 75 years old had a significantly higher fall risk compared to those aged 65–74 years (*p* = 0.001). Illiterate individuals had significantly higher Tw-FROP-Com scores than the other two groups (*p* = 0.022). Married participants had lower Tw-FROP-Com scores than unmarried participants (*p* = 0.007). Participants with a history of falling in the past year had significantly higher Tw-FROP-Com scores than those without a history of falling (*p* < 0.001). However, no significant difference was observed between participants living with others and those being cared for by others.

Among the frailty phenotypes, all five components—weakness, slowness, exhaustion, weight loss, and low physical activity—were significantly and positively associated with Tw-FROP-Com scores. We found that 18.7% of the 375 participants were classified as frail. Additionally, the comparison of characteristics between participants with and without a history of falls is presented in Table 2. Participants with a history of falls were significantly more likely to exhibit weakness (*p* = 0.014), slowness (*p* = 0.003), and a tendency toward frailty (*p* = 0.018).

**Table 2:** Univariate analysis of basic demographic characteristics among participants with and without falls (*n* = 425).

**Variables**	***N* (%)**	**Non-faller, *n* (%)**	**Faller, *n* (%)**	***p*-value**
**Gender**				
Male	102 (24.0)	72 (25.2)	30 (21.6)	0.489
Female	323 (76.0)	214 (74.8)	109 (78.4)	
**Age**				
65–74 years old	211 (49.6)	142 (49.7)	69 (49.6)	1.000
≥75 years old	214 (50.4)	144 (50.3)	70 (50.4)	
**Educational level**				
Illiterate	97 (23.7)	68 (24.5)	29 (22.0)	0.807
Elementary school	190 (46.5)	126 (45.5)	64 (48.5)	
Junior high school and higher	122 (29.8)	83 (30.0)	39 (29.5)	
**Race**				
Hokkien	354 (85.1)	233 (83.8)	21 (87.7)	0.370
Non-Hokkien	62 (14.9)	45 (16.2)	17 (12.3)	
**Marital status^a^**				
With a spouse	233 (55.7)	160 (56.7)	73 (53.7)	0.628
Without a spouse	185 (44.3)	122 (43.3)	63 (46.3)	
**Live with others or not**				
Live with others	355 (83.9)	239 (83.9)	113 (84.1)	1.000
Live alone	68 (16.1)	46 (16.1)	22 (15.9)	
**Caregivers**				
Cared by others	251 (59.6)	171 (60.4)	80 (58.0)	0.707
Cared by themselves	170 (40.4)	112 (39.6)	58 (42.0)	
**Frailty component**				
** *Weakness n* = (*411*)**				
Yes	197 (47.6)	121 (43.2)	86 (56.7)	0.014^*^
No	217 (52.4)	159 (56.8)	58 (43.3)	
** *Slowness* (*n = 409*)**				
Yes	159 (38.9)	93 (33.7)	66 (49.6)	0.003^**^
No	250 (61.1)	183 (66.3)	67 (50.4)	
** *Exhaustion* (*n = 408*)**				
Yes	105 (25.7)	73 (26.4)	32 (24.2)	0.722
No	303 (74.3)	203 (73.6)	100 (75.8)	
** *Weight loss* (*n = 395*)**				
Yes	18 (4.6)	257 (96.3)	120 (93.8)	0.390
No	377 (95.4)	10 (3.7)	8 (6.3)	
** *Low activity* (*n = 420*)**				
Yes	84 (20.0)	54 (19.1)	30 (21.9)	0.585
No	336 (80.0)	229 (80.9)	54 (19.1)	
**Frailty status^b^ (*n* = 375)**				
Yes	70 (18.7)	39 (15.2)	31 (26.1)	0.018^*^
No	305 (81.3)	217 (84.8)	88 (73.9)	

^a^“With a spouse” includes married status; “Without a spouse” includes single, divorced, and widowed. ^b^Frailty status was assessed based on Fried’s phenotype criteria. Participants meeting ≥3 of 5 criteria were classified as frail; those with 0–2 were non-frail. Chi-square tests were used for categorical comparisons; Yates’ continuity correction was applied to 2 × 2 tables when appropriate. ^*^p < 0.05, ^**^p < 0.01, ^***^p < 0.001.

**Figure 1:**
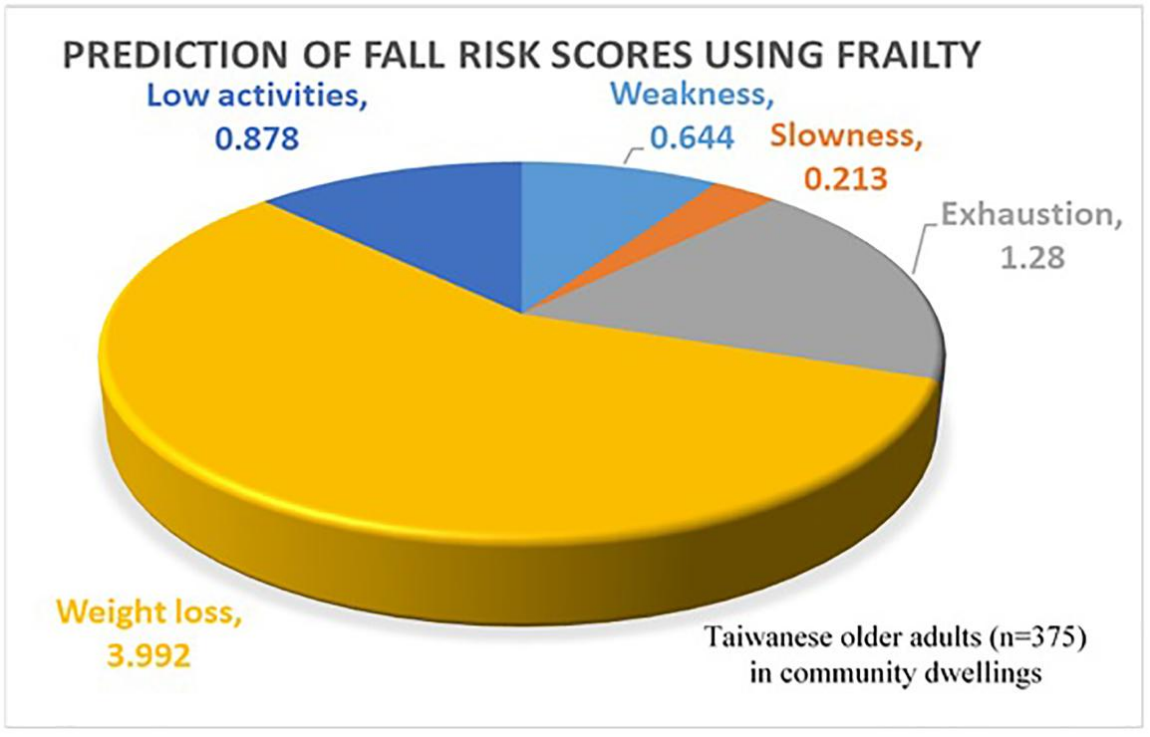
Distribution of fall risk scores predicted by individual frailty components among Taiwanese older adults (*n* = 375) living in community dwellings.

Using multivariable linear regression, we evaluated the association between fall risk scores (Tw-FROP-Com scores) and a history of falling, along with all components of frailty, after adjusting for age, education, and marital status. The results are presented in Table 3. Older adults who exhibited weakness (β = 0.644, 95% CI: 0.082–1.206), slowness (β = 0.213, 95% CI: 0.151–0.274), exhaustion (β = 1.280, 95% CI: 0.666–1.895), unintentional weight loss (β = 3.992, 95% CI: 2.781–5.203), and low physical activity (β = 0.878, 95% CI: 0.194–1.563) had significantly higher Tw-FROP-Com scores. Based on the B values, unintentional weight loss was the most influential factor for fall risk scores, followed by exhaustion, low physical activity, weakness, and slowness. The distribution of fall risk scores predicted by individual frailty components is illustrated in Figure 1.

**Table 3:** Multivariable linear regression analysis of fall risk scores based on frailty components (*n* = 375).

**Independent variables**	**β**	**Std. Error**	**95% CI**	***p*-value**
History of fall^#^	3.762	0.290	3.191–4.333	<0.001^***^
**Frailty component^#^**				
Weakness	0.644	0.286	0.082–1.206	0.025^*^
Slowness	0.213	0.031	0.151–0.274	<0.001^***^
Exhaustion	1.280	0.312	0.666–1.895	<0.001^***^
Weight loss	3.992	0.616	2.781–5.203	<0.001^***^
Low activities	0.878	0.348	0.194–1.563	0.009^**^
**Model R^2^**	0.552			

This model was adjusted for covariates including age, education, and marital status. β represents the coefficient of each variable. The multivariable linear regression formula was: Fall Risk = Fall History + Frailty Components + Age + Education + Marital Status. ^#^History of fall and frailty components are binary categorical variables (Yes/No), with “No” as the reference group. ^*^p < 0.05, ^**^p < 0.01, ^***^p < 0.001.

Frailty was treated as a dichotomous variable (Yes/No) in Table 4, whereas the model in Table 3 included five individual components. The results of the multivariable linear regression analysis for fall risk are presented in Table 4. Older adults with a history of falling had significantly higher fall risk scores than those without a history of falling (β = 3.886, 95% CI: 3.281–4.491). Additionally, frail older adults had higher fall risk scores compared to non-frail older adults (β = 3.886, 95% CI: 3.158–4.615).

**Table 4:** Multivariable linear regression analysis of the association of fall risk and frailty as well as fall history (*n* = 375).

**Independent variables**	**β**	**Std. Error**	**95% CI**	***p*-value**
History of fall^#^	3.886	0.370	3.281–4.491	<0.001^***^
Frailty status^#^	3.886	0.307	3.158–4.615	<0.001^***^
**Model R^2^**	0.484			

This model was adjusted for age, education, and marital status. The multivariable linear regression formula was: Fall Risk = Fall History + Frailty Status + Age + Education + Marital Status. Frailty status was a binary categorical variable (“frail” vs. “non-frail”). ^#^History of fall and frailty status are binary categorical variables (Yes/No), with “No” as the reference group. ^*^p < 0.05, ^**^p < 0.01, ^***^p < 0.001.

The only difference between the models in Tables 3 and 4 was how frailty was measured. The model in Table 3 assessed frailty as five separate components, whereas the model in Table 4 treated it as a dichotomous Yes/No variable. Based on the R² values of the multivariable linear regression models, the R² values for Tables 3 and 4 were 0.552 and 0.484, respectively. This finding suggests that evaluating individual frailty components predicts fall risk more effectively than using a dichotomous frailty measure, with a 6.8% increase in predictive accuracy.

Furthermore, we conducted a logistic regression analysis by categorizing the Tw-FROP-Com scores into two groups: the mild fall risk group (0–11) and the moderate fall risk group (12–60). We performed both crude odds ratio analyses and analyses adjusted for age, education, and marital status. Table 5 presents the association between fall history and each individual frailty component, evaluating their relationship with the fall risk groups. Older adults who exhibited weakness (aOR = 9.285, 95% CI: 2.672–32.262), slowness (aOR = 0.551, 95% CI: 0.163–1.860), exhaustion (aOR = 3.368, 95% CI: 1.063–10.673), unintentional weight loss (aOR = 23.843, 95% CI: 5.172–109.904), and low physical activity (aOR = 3.935, 95% CI: 1.263–12.261) had a higher likelihood of being in the moderate fall risk group.

**Table 5:** Multivariate logistic regression analysis of the association between fall history, frailty components and falls risk grading (*n* = 375).

**Independent variables**	**Model 1**			**Model 2**		
	**Crude OR**	**95% CI**	***p*-value**	**Adjusted OR**	**95% CI**	***p*-value**
History of fall^#^	6.339	2.569–15.642	<0.001^***^	9.285	2.672–32.262	<0.001^***^
**Frailty component^#^**						
Weakness	2.883	1.175–7.077	0.021^*^	0.551	0.163–1.860	0.337
Slowness	12.877	3.780–43.869	<0.001^***^	4.939	1.222–19.971	0.025^*^
Exhaustion	2.834	1.246–6.443	0.013^*^	3.368	1.063–10.673	0.039^*^
Weight loss	16.000	5.601–45.703	<0.001^***^	23.843	5.172–109.904	<0.001^***^
Low activities	3.307	1.304–7.073	0.010^*^	3.935	1.263–12.261	0.018
				Nagelkerke R^2^ = 0.459		

Falls risk grading was divided into two categories: mild and moderate risk, with the mild risk group serving as the reference category. In Model 1, crude odds ratios (ORs) were calculated for each frailty component individually. Model 2 was adjusted for age, education, marital status, and history of falling. ^#^History of fall and frailty components are binary categorical variables (Yes/No), with “No” as the reference group. ^*^p < 0.05, ^**^p < 0.01, ^***^p < 0.001.

The results of the multivariable logistic regression model for fall risk grading are shown in Table 6. Older adults with a history of falling had a significantly higher fall risk than those without a history of falling (aOR = 13.097, 95% CI: 4.117–41.667). Additionally, frail older adults had a significantly higher fall risk compared to non-frail older adults (aOR = 15.510, 95% CI: 4.526–53.153). According to the Nagelkerke R², the values for Tables 5 and 6 were 0.459 and 0.335, respectively. This finding suggests that frailty components predict fall risk grading more effectively than the dichotomous frailty measure (Yes/No), with a 12.4% improvement in predictive accuracy.

**Table 6:** Multivariate logistic regression analysis of the association between fall history, frailty and falls risk grading (*n* = 375).

**Independent variables**	**Model 1**			**Model 2**		
	**Crude OR**	**95% CI**	***p*-value**	**Adjusted OR**	**95% CI**	***p*-value**
History of fall^#^	6.339	2.569–15.642	<0.001^***^	13.097	4.117–41.667	<0.001^***^
Frailty status^#^	6.729	2.719–16.653	<0.001^***^	15.51	4.526–53.153	<0.001^***^
				Nagelkerke R^2^ = 0.335		

Falls risk grading was divided into two categories: mild and moderate risk, with the mild risk group serving as the reference category. In Model 1, crude odds ratios (ORs) were calculated for each variable individually. Model 2 was adjusted for age, education, and marital status. Frailty status was a binary categorical variable (“frail” vs. “non-frail”). ^#^History of fall and frailty status are binary categorical variables (Yes/No), with “No” as the reference group. ^*^p < 0.05, ^**^p < 0.01, ^***^p < 0.001.

## DISCUSSION

To the best of our knowledge, this study is the first to evaluate the association between frailty components and fall risk scores, as measured by the Tw-FROP-Com assessment tool, among community-dwelling older adults in Taiwan. Our findings indicate that frailty components account for more than 50% of the variation in fall risk scores among individuals with a history of falls. Older adults experiencing unintentional weight loss, weakness, exhaustion, slowness, and low physical activity had significantly higher Tw-FROP-Com scores after adjusting for sociodemographic factors and fall history. As expected, frail older adults exhibited significantly higher fall risk scores than non-frail individuals. Among the five frailty components, unintentional weight loss emerged as the most influential factor affecting fall risk. Treating frailty as five distinct components provided a more precise prediction of fall risk than using a dichotomous frailty measure (Yes/No). Unintentional weight loss was identified as a critical intrinsic factor, highlighting the need for a differentiated approach to the frailty phenotype among the oldest old, taking into account the specific influence of its components [15]. Each individual indicator can be used to screen older adults at high risk of falls, enabling early detection and timely intervention. Based on the specific scores of each frailty component, tailored fall prevention strategies can be implemented, such as weight management, dietary recommendations, and exercises to improve muscular strength and endurance. These targeted interventions aim to effectively reduce the risk of falls among the elderly population.

Since unintentional weight loss emerged as a key factor in frailty associated with fall risk among older adults, effective weight management and regular monitoring are critical components of fall prevention strategies. Despite the various criteria used to define frailty, weight loss has consistently been a significant predictor of future falls among both community-dwelling [29] and hospitalized older adults [30]. However, previous studies primarily used Fried frailty status (Yes/No) as an outcome variable rather than examining frailty through its five distinct phenotypes. Moreover, no existing studies have assessed fall risk among community-dwelling older adults using both the Fried frailty index and the Tw-FROP-Com. In contrast, our study distinguished the five frailty phenotypes and assessed the overall fall risk. Additionally, the Tw-FROP-Com proved to be a practical and efficient tool, requiring no supplementary instruments to comprehensively assess the 13 fall risk domains. Its simplicity makes it highly applicable in both clinical and community settings for the early identification of older adults at risk of falling. Numerous studies have demonstrated a connection between frailty status and a history of falls [14, 21, 31]. Our findings offer novel insights compared to previous studies. For example, a study conducted in Brazil interviewed 1,413 older adults to assess the association between frailty and falls. The frailty components associated with fall risk included: (1) reduced grip strength (no falls: 21.8%; falls: 31.5%; relative risk (RR) = 1.44; *p* = 0.003) and (2) exhaustion (no falls: 7.6%; falls: 14.7%; RR = 1.93; *p* = 0.003) [32]. In our study, unintentional weight loss was the most powerful predictor of fall risk among the five frailty components. This finding suggests that the contribution of frailty components to fall risk may vary by race, age, and other physical conditions.

Unintentional weight loss is commonly associated with malignant diseases, psychiatric disorders, gastrointestinal conditions, endocrine disorders, and cardiovascular diseases [33]. Many older patients with unintentional weight loss also report experiencing concomitant malnutrition [34]. Numerous studies have shown that both unintentional and intentional weight loss are unfavorably associated with increased mortality, morbidity, in-hospital complications, and reduced functional capacity for independent living [33, 35–37]. Rapid unintentional weight loss in older adults often indicates underlying diseases and accelerates age-related muscle loss. Weight loss can result from various physical, psychological, and social conditions, as well as age-related physiological changes. However, up to one-fourth of patients may have no identifiable cause [33].

Malnutrition is a key contributing factor to both weight loss and frailty in older adults. Studies examining the effects of malnutrition and falls among elderly inpatients have identified several risk factors [38–40]. Poor nutrition reliably predicts falls in hospitalized older patients, with malnourished individuals exhibiting higher fall rates [39]. In clinical practice, interventions such as nutritional screening and assessment should be implemented to prevent these avoidable falls. Poorly nourished participants had an elevated risk of self-reported falls over six months (RR = 1.5, 95% CI: 1.0–2.5, *p* = 0.03) [40]. The relationship between malnutrition and body weight loss is inextricably linked [38]. Older adults experiencing body weight loss may have an increased risk of falls due to chronic malnutrition. Malnutrition is often a subtle underlying condition, whereas subsequent body weight loss is a visible clinical manifestation. Evaluating body weight loss is more accessible and intuitive for clinicians, enabling them to quickly identify patients at high risk of falling. However, clinicians often face challenges in diagnosing weight loss in older adults.

In most cases, a diagnosis can only be made after a thorough patient history, physical examination, and basic laboratory evaluation, followed by the prescription of nutritional supplements [41]. If the initial evaluation is unremarkable, malnutrition should be considered a contributing factor [33]. Malnutrition screening tools are effective in identifying older adults at risk, enabling early prevention. Additionally, several quantitative malnutrition screening tools have been developed and evaluated for their effectiveness [42, 43]. A review of 74 papers covering 119 validation studies on 34 malnutrition screening tools used in older adults across various settings was conducted. The validation results varied considerably across tools, studies, and settings [43]. Therefore, tools for assessing malnutrition should be carefully selected based on the age of the subjects, the target population, and the intended setting.

Identifying and addressing any underlying causes is the primary goal in managing weight loss [33]. Treatment for unintentional weight loss often requires improving access to adequate nutrition [33]. Therefore, several essential non-pharmacological strategies can be implemented to prevent or manage malnutrition and promote adequate food intake. Additionally, other factors contributing to poor diet quality should be considered, including poverty, poor dental health, difficulties with chewing or swallowing, stress, and emotional distress [33].

### Limitation

Several limitations of this study should be acknowledged. First, the relatively small sample size may have led to inconclusive results, potentially affecting the validity of the findings. Second, the cross-sectional study design limits the ability to establish temporality between exposure and outcome. To clarify causal relationships, explanatory research designs are necessary, as the observed associations may be difficult to interpret. Third, potential biases in our research setting may include recording bias, responder bias, recall bias, social desirability bias, and interviewer bias. These biases are often difficult to avoid when evaluating fall history among older adults. Additionally, assessing psychological conditions related to weight loss, such as depression, could have provided more comprehensive insights. Therefore, future studies should adopt a longitudinal design to observe and analyze these variables over time. Implementing random sampling in participant recruitment would also improve the generalizability of the findings. This approach would better capture the relationship between weight changes and fall risk.

## CONCLUSIONS

Unintentional weight loss has been identified as the most influential frailty component in predicting fall risk among older adults in Taiwanese community settings. The five individual frailty phenotypes predict fall risk more effectively than overall frailty status. However, the relationship between frailty, its components, and fall risk is complex and warrants further investigation. Our findings highlight the need for future studies to prioritize longer follow-up cohort designs to confirm the causal relationship between specific frailty phenotypes and fall risk among community-dwelling older adults. A deeper understanding of these associations could inform more targeted and effective fall prevention strategies for this population.

## MATERIALS AND METHODS

### Participants

This cross-sectional study was conducted in Keelung City, a metropolitan area in northern Taiwan. A total of 375 participants were enrolled in an Elderly Fall Prevention Program (Supplementary Table 1). The study used the Taiwan version of the Falls Risk for Older People in the Community (Tw-FROP-Com), established through a standardized translation procedure, to identify older adults at risk of falling. The inclusion criteria were as follows: older adults aged ≥65 years who met at least one of the following conditions: (1) experienced a fall within the past 12 months or (2) exhibited a fear of falling.

### Frailty phenotypes

We modified the definition of frailty status based on the frailty syndrome proposed by the Cardiovascular Health Study (CHS) [16]. While traits such as “weakness,” “slowness,” and “exhaustion” remained unchanged, the definitions of “weight loss” and “low physical activity” were adapted to better suit the elderly population in Taiwan. The definitions and criteria for each frailty phenotype are detailed below:

- Weakness: Grip strength was measured as the mean of three measurements from the dominant hand. Participants with grip strength values below 26 kg for males and 18 kg for females met the criteria for weakness [44].
- Slowness: Slowness was defined as the time required to walk 5 meters. Participants with a walking speed of less than 0.8 m/sec were categorized as slow [44].
- Exhaustion: This was assessed using two self-reported statements: (1) “I felt that everything I did was an effort” and (2) “I could not get going” [45]. Participants rated how often they experienced these feelings in the previous week:
  0: Rarely or none of the time (0–1 day)
  1: Some or a little of the time (1–2 days)
  2: A moderate amount of the time (3–4 days)
  3: Most of the time
  Participants scoring “2” or “3” on either statement were categorized as frail under the exhaustion criterion [16].
- Weight loss: Unintentional weight loss was defined as a loss of more than 3 kg or greater than 5% of body weight in the preceding year [46].
- Low physical activity: Energy expenditure was assessed using the International Physical Activity Questionnaire (IPAQ) Short Form, specifically the Taiwan IPAQ for older adults. Physical activity during the previous week was quantified. Males with weekly energy expenditure below 2375.6 kcal and females below 2432.4 kcal—representing the lowest 20% of participants—met the criterion for low physical activity [46].

Participants were classified as robust if they met none of the criteria, prefrail if they met 1–2 criteria, and frail if they met ≥3 criteria.

**Table:** 

**Frailty component**	**Definition**
**Weakness**	Males: Grip strength <26 kg
	Females: Grip strength <18 kg
**Slowness**	Walking 5 meters at a speed of ≤0.8 m/sec
**Exhaustion**	Participants reporting feeling (1) “I felt that everything I did was an effort” or (2) “I could not get going” for more than 3 days in the previous week met the criterion.
**Weight loss**	Unintentional weight loss >3 kg or greater than 5% of body weight in the preceding year
**Low physical activity**	Males: Energy expenditure <2375.6 Kcals per week
	Females: Energy expenditure <2432.4 Kcals per week

### Fall risk assessment

Fall risk was assessed using the community-based comprehensive fall risk assessment tool, the Taiwanese version of the Falls Risk for Older People in the Community (Tw-FROP-Com). The FROP-Com demonstrated excellent reliability and moderate ability to predict falls. In a study evaluating its reliability, validity, and accuracy, the intra-class correlation coefficients for intra-rater and inter-rater reliability of the FROP-Com were 0.93 and 0.81, respectively [47]. For the Tw-FROP-Com, these values were 0.99 and 0.97 [48].

The Tw-FROP-Com consists of 28 items covering 13 identified risk factors for falls. These risk factors are summarized in the table below:

**Table:** 

**Tw-FROP-Com items**	**Description**	**Scoring**
History of falls	History of falls and fall-related injuries	0–3 points
Medications	Use of sedatives, anticoagulants, or other medications that elevate fall risk	0–3 points
Medical conditions	Conditions such as arthritis, Parkinson’s disease, stroke, cardiac conditions, diabetes, osteoporosis	0–3 points
Sensory loss	Vision and somatosensory impairments	0–1 points
Feet and footwear	Foot-related issues and inappropriate footwear	0–1 points
Cognitive status	Cognitive impairment or decline	0–3 points
Continence	Issues with urinary or fecal continence	0–1 points
Nutritional status	Recent weight changes or malnutrition	0–3 points
Environment	Hazards such as poor lighting, steps, or uneven surfaces	0–3 points
Functional behaviour	Fear of falling or risk-taking behaviors	0–3 points
Function	Difficulty performing activities of daily living	0–3 points
Balance	Unsafe or irregular walking patterns that increase fall risk	0–3 points
Gait/Physical Activity	Low levels of physical activity, quantified as energy expenditure below specified thresholds	0–3 points

Each item was scored on a scale of 0–1 or 0–3, indicating the severity of individual risk factors and the overall fall risk. In our analysis, fall risk scores were categorized into two subgroups: mild fall risk (0–11) and moderate fall risk (12–60).

### Statistical analysis

Descriptive statistics for participant characteristics were summarized using IBM SPSS Statistics (Version 28) [49]. Means and standard deviations were calculated for continuous variables. For group comparisons, t-tests were applied for two groups, and one-way ANOVA for three or more groups. Multivariate linear regression was performed to examine the association between frailty phenotypes/status and the Tw-FROP-Com score. Additionally, multivariate logistic regression was used to evaluate the relationship between frailty phenotypes/status and the Tw-FROP-Com risk grading. A *p*-value of < 0.05 was considered statistically significant.

## Supplementary Materials

Supplementary Table 1
